# Metal nanoparticles functionalized with nutraceutical Kaempferitrin from edible *Crotalaria juncea*, exert potent antimicrobial and antibiofilm effects against Methicillin-resistant *Staphylococcus aureus*

**DOI:** 10.1038/s41598-022-11004-2

**Published:** 2022-04-29

**Authors:** Bhanuvalli R. Shamprasad, Robert Lotha, Saisubramanian Nagarajan, Arvind Sivasubramanian

**Affiliations:** 1 Department of Chemistry, School of Chemical and Biotechnology, SASTRA Deemed to Be University, Thanjavur, Tamil Nadu India; 2 Center for Research On Infectious Diseases, School of Chemical and Biotechnology, SASTRA Deemed to Be University, Thanjavur, Tamil Nadu India

**Keywords:** Nanobiotechnology, Biofilms

## Abstract

Kaempferitrin (KF), a flavonol glycoside, was isolated from the edible plant *Crotalaria juncea*. Optimization for the synthesis of silver (AgNPs) and copper (CuNPs) nanoparticles using *C. juncea* extract and kaempferitrin were attempted for the first time. A detailed study on size and stability analysis have been reported. Efficacy of KF@AgNPs and KF@CuNPs against biofilm formation and planktonic mode of growth on methicillin-resistant *Staphylococcus aureus* (MRSA) along with possible mechanisms has been explored. Release of Cu(II) upon prolonged treatment with KF@CuNPs in the presence of MRSA was quantified through Alizarin red test, indicating the antibacterial effect is initiated by the CuNPs itself. Time kill curve depicted both the NPs have similar kill kinetics to curtail the pathogen and imaging with Crystal violet assay, Fluorescent live dead imaging and SEM analysis revealed a 60% reduction in biofilm formation at the Sub-MIC concentration of KF@AgNPs and KF@CuNPs. Furthermore, the membrane permeability and cell surface hydrophobicity were altered in the presence of both the NPs. The colony count from the in vivo infection zebrafish model in the treatment group showed a decline of > 1.8 fold for KF@AgNPs and > two fold for KF@CuNPs. Toxicity studies did not reveal any abnormality in liver and brain enzyme levels. Liver morphology images show no severe cytological alterations when treated with KF@AgNPs and were almost similar to the normal liver. Thus, KF@AgNPs was nontoxic and caused significant reduction in biofilm formation in MRSA, also reduced bacterial bioburden in the infected zebrafish, which has the potential to be explored in higher animal models.

## Introduction

*Staphylococcus aureus* is an opportunistic pathogen that is responsible for a wide range of clinical infection and is acknowledged as the key nosocomial pathogen causing significant mortality and morbidity^1–3^. The progress of Methicillin-resistant *Staphylococcus aureus* (MRSA) both as community acquired (CA)-MRSA and hospital acquired (HA)-MRSA is in part due to its ability to adhere on the abiotic /biotic surfaces and its propensity to form biofilms^4^. As per the Centers for Disease Control and Prevention (CDC)-U.S. (2017) report, every year roughly 119,000 people were infected by MRSA that results in almost 20,000 deaths. Another survey by the Indian Network for Surveillance of Antimicrobial Resistance (INSAR) group revealed a high prevalence (~ 40%) of Methicillin-resistant *Staphylococcus aureus* (MRSA) over a two-year period across 15 different tertiary care centers in India^5^. Increase in resistance to antibiotics has put forward a research problem for future therapeutic strategies to combat the drug resistance in many pathogenic strains. Therapeutic properties from the plant source have been long explored to treat many common health ailments and disease with negligible side effects^6,7^. Various plant phytochemicals are reported to possess antimicrobial properties, including biofilm inhibition^8,9^.

In the recent years metallic nanoparticles (MNPs) ranging from 1 to 100 nm are a particularly important class of nanomaterials with their unique distinctive properties, due to their large surface area to volume ratio are used in diverse applications such as drug delivery, anticancer agents, antifungal, antimicrobial^10^. It has been long understood that plants can reduce metal ions^11^ by the presence of phytochemicals which serve both as a stabilizers and natural reductants^12^ that inhibit the agglomeration/aggregation of MNPs by non-hazardous process^13^. Plant extracts/secondary metabolites mediated synthesis of MNPs has gained popularity due to its low cost, simplicity, ease of scale-up, sustainability and eco-friendly nature^14^. In addition, when the plant extract or the secondary metabolites are capped with metallic nanoparticles the antimicrobial potential is immensely enhanced^15^. Secondary metabolites like flavonoids, terpenoid glycosides, fatty acids^16,17^, from plant sources have been used as a reducing and capping agent for the nanoparticle green synthesis. Silver and copper metals have been traditionally used in various forms as antimicrobial agents, and have inspired many groups in nanotechnology to synthesize antimicrobial metallic nanoparticles, through a greener method^18,19^. Also there are reports, which suggest that antimicrobial metal nanoparticles which were functionalized with phytochemicals/secondary metabolites, had a prominent role in reducing the microbial burden in surfaces and in vivo systems^20^.

*Crotalaria juncea* belongs to the family of *Fabaceae*, and is one of the major wild edible species^21^ reported to have antibacterial activity^22^. To our knowledge there are no reports regarding nano-therapeutic efficacy of *C. juncea* with noble metals regarding the antibacterial and antibiofilm activities. Herein, we report the nano-conjugation of *C. juncea* extract and its isolated secondary metabolite-Kaempferitrin (flavanol glycoside), to the elemental silver and copper respectively, with an objective to gain mechanistic insights into the antibiofilm potential; and test its toxicity and evaluate its antibacterial properties, in an in vivo zebrafish infection model.

## Materials and methods

### Plant collection and chromatography of the extract

The use of plant material in the study complies with relevant institutional, national, and international guidelines and legislation. The present study complies with the IUCN Policy Statement on Research Involving Species at Risk of Extinction and the Convention on the Trade in Endangered Species of Wild Fauna and Flora, and is not in the red list of IUCN. The aerial (leaves and stem) parts of *C. juncea* were collected at Wokha, Nagaland, India, *circa* March 2018. The plant was authenticated by Dr. Ravichandran N, Centre for Advanced Research in Indian System of Medicine (CARISM), SASTRA Deemed to be University, Thanjavur, India. A voucher specimen (CARISM 00147) was deposited in the herbarium of CARISM, SASTRA Deemed to be University, Thanjavur, India. The plant parts (1 kg) were dried at ambient temperatures, pulverized to a powder, and percolated with MeOH. After filtration, the extract was concentrated in a rotary evaporator and further lyophilized to obtain a residue, CJM (45 g).

CJM (20 g) was taken for column chromatography with silica gel (100–200 mesh) packed in a glass column (1 cm in diameter, 15 cm in length) and eluted with CHCl_3_-MeOH (2,8,15,25 and 30%), and MeOH successively. Two fractions eluted at 25% and 30% CHCl_3_-MeOH were pooled together and subsequently run in Sephadex LH-20 chromatography with an elution system of 30% CHCl_3_-MeOH.

### Synthesis of biogenic silver and copper nanoparticles

#### Optimization for the synthesis of *C. juncea* extract capped AgNPs / Kaempferitrin@AgNPs

The effects of various influencing factors were considered for the synthesis of biogenic AgNPs. Initially two of the factors were kept constant and the desired factor was varied at different levels. (i) *C. juncea* extracts (2, 3, 4 mg/mL)/ Kaempferitrin (0.5, 1 and 1.5 mg/mL) (ii) Silver nitrate (SN) molarity: 1, 2 and 3 mM, (iii) Sunlight exposure time (hrs or min). OD was noted using UV–Vis spectroscopy at a regular time interval of 30, 60 and 120 min in the case of extract and 60, 90 and 120 min for Kaempferitrin (Fig. S1). The as-prepared, optimized biogenic AgNPs were characterized and continued for further studies.

#### Optimization for the synthesis of *C. juncea* extract capped CuNPs/Kaempferitrin@CuNPs

For CuNPs synthesis, i) *C. juncea* extracts (2, 3, 4 mg/mL)/ Kaempferitrin (0.5, 0.75 and 1 mg/mL) ii) Cupric acetate monohydrate (CAM) molarity: 1, 2 and 3 mM iii) Reducing agents (RA) combination: Ammonium hydroxide (NH_4_OH) and Hydrazine hydrate (H_6_N_2_O) were used at different volume as RA1; 5 and 10µL/mL, RA2; 10 and 20 µL/mL and RA3; 15 and 30 µL/mL respectively (Fig. S1). Synthesized CuNPs by the optimization were characterized and retained for further studies.

### Characterization of nanoparticles functionalized with secondary metabolites

UV–visible spectroscopy was employed for analysis of bio-reduction of silver nitrate/Cupric acetate monohydrate to AgNPs/CuNPs using (Lambda 25, PerkinElmer, Waltham, MA) by monitoring the spectra from 200 to 800 nm. X-ray diffraction (XRD) patterns were recorded using Rigaku Ultima III diffractometer (Rigaku, Tokyo, Japan) with Cu-Ka radiation in the 2-h range from 10 to 80 kV. The dispersed AgNPs/CuNPs were taken in polystyrene cuvette and subjected to the particle size analysis and zeta potential, done with Laser Diffractometry coupled ZetaSizer Nano-series (ZS-90 Red, Malvern Instruments, Malvern, England). The surface morphology of AgNPs/CuNPs was analyzed using the Field Emission Transmission Electron Microscope (FE-TEM 2701F, JEOL, Tokyo, Japan).

### Stability studies for AgNPs and CuNPs

To analyze the stability and agglomeration of synthesized nanoparticles (KF@AgNPs and KF@CuNPs), the nanoparticles synthesized at optimized conditions were prepared in bulk volume and stored in a dark place, with all NPs of equal volumes. Each sample was analyzed by UV–visible spectrum (200–800 nm) at various time points, followed by Zeta potential and Zeta sizer analysis for confirmation.

### Antimicrobial studies

#### Bacterial strains

Methicillin resistant *Staphylococcus aureus* ATCC43300 was employed in the present study. The strain was maintained as 15% glycerol stocks at -80 ^o^C. The strains were subcultured from a glycerol stock onto LBA/TSA plates for regular use. The isolated MRSA colony was inoculated into LB broth.

#### Screening for antibacterial activity

Minimum inhibitory concentration (MIC) and minimum bactericidal concentration (MBC) was evaluated for bacteriostatic and bactericidal effect of CJE@AgNPs/CuNPs and KF@AgNPs/CuNPs, as reported earlier using micro broth two fold dilution method was used^23,24^.

#### Time kill kinetic assay curve

Time kill assay was performed to evaluate bactericidal potential of biogenic AgNPs/CuNPs. Briefly, overnight culture of MRSA was diluted to 0.05 OD in sterile broth and cells were allowed to grow till 0.1 OD (10^6^ cells), measuring the optical density at 595 nm. At this time point, treatment was initiated with 1X MBC of AgNPs/CuNPs. Samples were retrieved at different time points (0, 1, 2, 3, 4, 5 & 24 h), serially diluted and plated onto Nutrient agar plates and plate counts were determined for both untreated and treated samples^25^.

#### Alizarin Red-Q assay

The amount of copper ions that are released from CuNPs in the absence and presence of MRSA is quantified as copper ions complexed with Alizarin red at fixed absorbance of 510 nm^26^. Overnight culture was diluted to 0.3 OD and were treated with CuNPs at their 1X MIC. Copper ions treated sterile media and media without copper ions were maintained as controls. Pelleted cells treated with CuNPs are resuspended in 1 mL of 0.2 M sodium acetate buffer pH 5.0. Alizarin Red-Q (200 µl) was added to samples and absorbance at 510 nm of these samples was recorded at different time intervals.

### Studies on biofilm inhibition

#### Crystal violet (CV) staining

MRSA overnight grown culture was diluted to 0.05 OD and the bacteria were allowed to attach onto the surface of sterilized glass slides in a sterile petri plate. After allowing the cells to attach on glass surface for 5–10 min, the slides were gently immersed into sterile Brain Heart Infusion (BHI) broth with or without KF@AgNPs and KF@CuNPs and allowed to incubate for 24 h. Post incubation, the slide was carefully taken out using forceps and the unbounded planktonic cells were removed by sterile PBS wash thrice and were air dried. The slides were stained using CV (0.1%) for 20-25 min. Excess CV dye was washed away using double distilled H_2_O and stained slides were dried for 40 min in an oven. Biofilm formed on the slides were imaged using (Nikon Eclipse Ni-U, Japan) optical microscopy^27^.

#### Fluorescent live/dead imaging

For fluorescence live/dead imaging of biofilm, bacteria were allowed to attach on the surface of the sterile glass slides placed within Petri plates, the slides were immersed using sterile BHI broth. Different groups were allocated untreated and KF@AgNPs and KF@CuNPs treated independently. Untreated groups were maintained as control. After 24 h of incubation, the slides were washed gently with sterile PBS and stained using a mixture of Fluorescein diacetate and propidium iodide @ 1:1 ratio. The image was captured using a Nikon fluorescence microscope (Nikon Eclipse Ni-U, Japan)^28^.

#### SEM imaging

For SEM imaging studies, overnight culture of MRSA was diluted to an OD of 0.05 and allowed to attach on the surface of sterile cover glass, placed within microtiter plate (24 well), The coverglass was immersed in sterile BHI broth and kept for 24 h incubation, sterile PBS was used to wash the unbound cells and 2% glutaraldehyde was introduced as fixative. Further, washed with sterile PBS and air-dried. The biofilms, which were formed on the surface of the cover glass, were subjected to dehydration using a series of ethanol wash (50%-100%) for each 10 min. Subsequently, the biofilms were air-dried and sputter coated with platinum, image was captured by FE- SEM, JEOL 6701F (Tokyo, Japan)^29^.

### Cell membrane permeability assay

Mid-log phase culture of MRSA was centrifuged and the pelleted cells were washed with sterile PBS twice and then re-suspended with PBS of equal volume. KF@AgNPs and KF@CuNPs and CTAB 0.2 mmol/l along with propidium iodide 30 µmol/1 were added and incubated at 37 °C for 2 h. Fluorescence intensity of PI was measured by using (Jasco FP-8500, Jasco, Tokyo, Japan) spectrofluorimeter. Permeability index was determined by calculating the fluorescence ratio between cells without and treated with CTAB. The fluorescence normalization was carried out by subtracting fluorescence of treated cells with untreated cells^30^.

### BATH assay

Bacterial cell surface hydrophobicity was evaluated by determining the ability of cells to partition from the aqueous phase to Hexadecane. To determine the changes in surface hydrophobicity when treated with KF@AgNPs and KF@CuNPs hexadecane-aqueous partition method as reported earlier^31^ was adopted. After vortexing the mixture containing MRSA cells, hexadecane and PBS, final absorbance is taken at OD_530_ from the aqueous phase.

### Reactive Oxygen species (ROS) assay

Production of ROS after treatment with Sub-MIC of KF@AgNPs and KF@CuNPs in MRSA were determined using fluorophore Dichloro-dihydro-fluorescein diacetate (DCFH-DA) which gets reduced to dichlorofluorescein in a ROS mediated formation, which was quantified using a fluorescence spectrophotometer (JASCO FP-8500, JASCO, Tokyo, Japan) at Ex 485 nm and Em 538 nm^32^.

### Studies on in vivo model system

#### Ethical Statement

The study was performed in compliance with all relevant ethical laws and guidelines in India. The CPCSEA guidelines for laboratory animal facilities (Central Act 26 of 1982) in India were adhered to in all the in vivo experiments. The experimental protocols were approved by the Institutional Animal Ethics Committee (CPCSEA-493/SASTRA/IAEC/RPP) of SASTRA Deemed University, India . Reporting of all experimental procedures complied with recommendations in ARRIVE guidelines.

#### Zebrafish toxicity study design

Adult zebrafish (*Danio rerio*) irrespective of sex, aged 2 months, measuring 4 to 5 cm in length, weighing approx. 300 mg, were purchased from a local aquarium in Thanjavur, Tamil Nadu, and India. Animal acclimatization was carried out following established protocols^33^. To check the effect of CuNPs on liver enzyme profiles of zebrafish, 5 fish each were exposed to the 1X MIC of the MNPs/Standard drug for 48 h. At the end of exposure (48 h), fish were anesthetized with ms-222 and euthanized by decapitation. The liver tissues were pooled from fish within each group and homogenized in an ice-cold buffer (Tris–HCl, 0.1 M, pH 7.4). The homogenate was centrifuged (10,000 × g, 10 min, 4 °C) and the supernatant was used for all analyses in triplicates. Using the supernatant liver, α- and β-carboxylesterase enzyme activities were estimated^34^, acetylcholinesterase (AChE) activity was measured by DNTB (5,5'-dithio-bis-[2-nitrobenzoic acid]) degradation^35^ Protein was estimated by the Lowry method^36^.

#### Histological study on zebrafish liver.

All fish from the treated and untreated group (n = 5) were euthanized in ice cold water at the end of the experiment before fixing in buffered 10% formalin solution for 24 h and later paraffin wax was used to embed. Sections of 5 μm thickness were cut and the thin sections of the tissue were stained by both eosin and hematoxylin for histological examination of the liver^37^.

#### Effect of KF@AgNPs and KF@CuNPs *on in-vivo* zebrafish infection model

Overnight grown MRSA was diluted to 0.05 OD with sterile media and 10 µl of culture was delivered through an intramuscular injection using a 3/10-cc U-100 insulin syringe with a 0.5-in-long, 29-gauge needle. The fish were grouped (n = 5) as infected treated and infected untreated. After 3 h post-infection, 10 µl of KF@AgNPs and KF@CuNPs at 1X MIC were delivered near the site of infection, grouped as treated^38^. The fishes were anesthetized with ms-222 after 24 h and euthanized by decapitation and the muscle tissues were collected by dissection, homogenized in sterile PBS; plated onto LB agar plates (serially diluted) to determine the microbial load in respective treatments.

### Statistical analysis

All experiments were performed thrice and data are presented as the mean ± standard deviation (SD). One-way analysis of variance (ANOVA), followed by a post hoc multiple comparisons (Tukey test) was done for grouped data analysis. Values were considered statistically significant at p < 0.05.

## Results and discussion

### Characterization of Kaempferitrin isolated from *Crotalaria juncea*

Kaempferitrin (42 mg) was isolated from *C . juncea* as an amorphous yellowish solid having a molecular formula of C_27_H_30_O_14_. The structure of kaempferitrin (Fig. S2–S3) was unequivocally ascertained using NMR spectroscopy^39^.

### Synthesis and optimization of biogenic silver and copper nanoparticles

#### *C. juncea* extract and Kaempferitrin capped AgNPs

The optimization studies for the synthesis of biogenic AgNPs was performed by determining its wavelength (380-450 nm) and Absorbance (OD) through UV–visible spectroscopy, taking into various physicochemical and environmental factors, later the optimized AgNPs size was analyzed; likewise the stability of each biogenic AgNPs was monitored at every time intervals (days). Two of the factors were kept constant, and the desired factor was varied at different concentrations/durations. For the synthesis of *C. juncea* spp. capped AgNPs, the extract concentration was taken between 2 and 4 mg/mL, and the silver-precursor (AgNO_3_) concentration was again taken in 0.5 mM in a range of 1-3 mM. The sunlight exposure time ranged between 30 min to 3 h. Thus, for CJE@AgNPs was optimized at CJE concentration of 2 mg/mL, AgNO_3_ of 1 mM and exposure time of 120 min (Table 1).

**Table 1:** Optimized variables for synthesis of Silver/Copper nanoparticles and their particle size.

*Silver nanoparticles*	*Crotalaria juncea*	Kaempferitrin
Concentration (mg/mL)	2	1
AgNO_3_ (mM)	1	2
Exposure (min)	120	90
**Particle size (nm)**	64	33

***Copper nanoparticles***		
Concentration (mg/mL)	3	1
Cupric acetate (mM)	2	2
NH_4_OH and H_6_N_2_O (µL/mL)	10 and 20	5 and 10
**Particle size (nm)**	92	56

**Figure 1:**
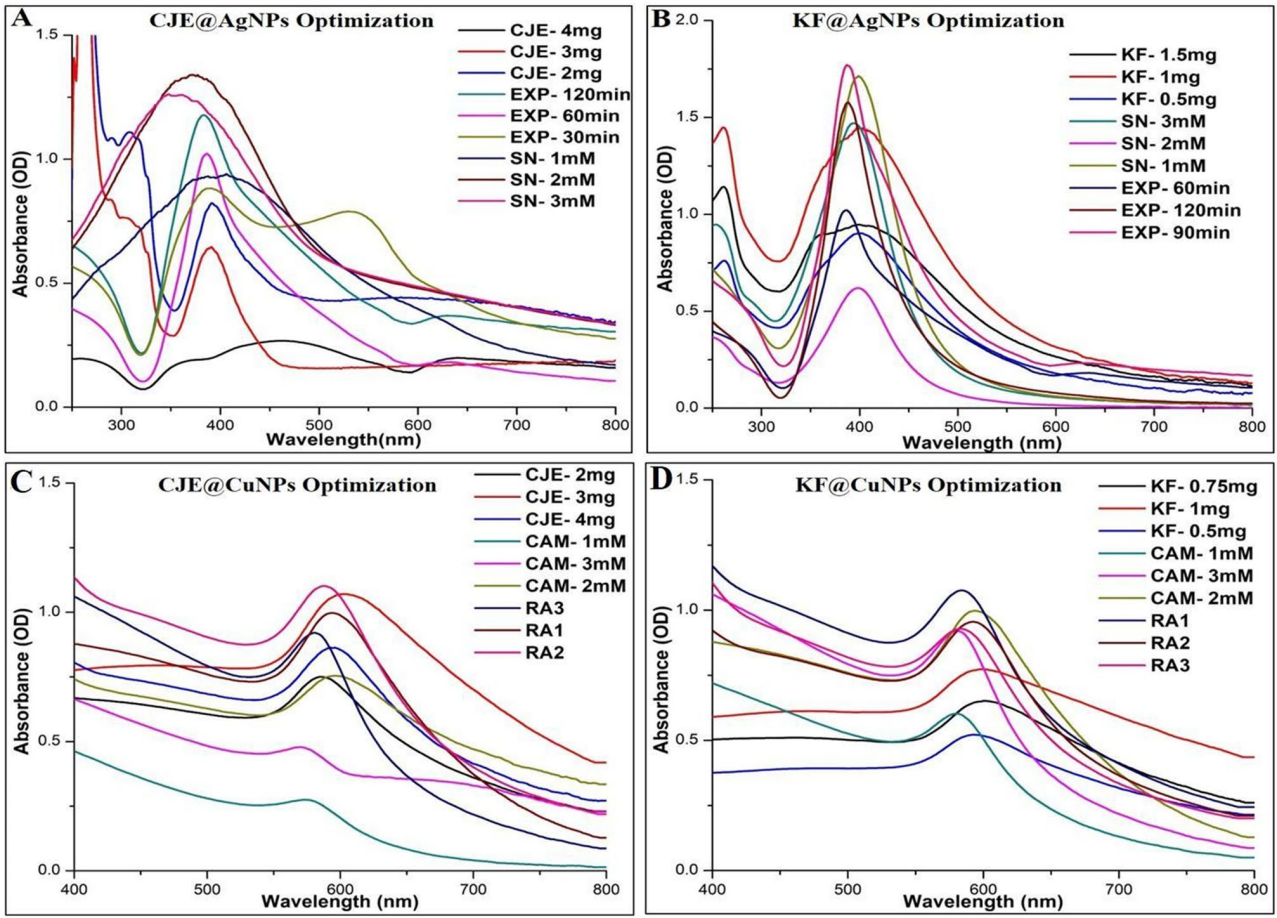
Optimization studies for the synthesis of *Crotalaria juncea* extract and Kaempferitrin capped AgNPs/CuNPs (**A**). CJE (2, 3 and 4 mg/mL) Vs SN (1 mM) and EXP (1 h); SN (1, 2 and 3 mM) Vs CJE (Optimized concentration) and EXP (1 h); EXP (1, 2 and 3 h) Vs CJE (Optimized concentration) and SN (optimized molarity). (B). Kaempferitrin (0.5, 1 and 1.5 (mg/mL) Vs SN (1 mM) & EXP (60 min); SN (1, 2 and 3 mM) Vs Kaempferitrin (Optimized concentration) & EXP (1 h)’ iii) EXP (60, 90 and 120 min) Vs Kaempferitrin (Optimized concentration) & SN (optimized molarity). (**C**). CJE (2, 3 and 4 mg/mL) Vs CAM (1 mM) and RA1 (µL/mL); CAM (1, 2 and 3 mM) Vs CJE (Optimized concentration) and RA1 (µL/mL); RA (1, 2 and 3 µL/mL) Vs CJE (Optimized concentration) and CJE (optimized molarity). (**D**). Kaempferitrin (0.5, 0.75 and 1 mg/mL) Vs CAM (1 mM) and RA1 (µL/mL);, CAM (1, 2 and 3 mM) Vs Kaempferitrin (Optimized concentration) and RA1 (µL/mL); RA (1, 2 and 3 µL/mL) Vs Kaempferitrin (Optimized concentration) and CAM (optimized molarity).

As for the optimization of AgNPs functionalized with kaempferitrin (KF), the KF concentration was taken between 0.5 and 1.5 mg/mL aliquots and the silver-precursor (AgNO_3_) concentration was again taken in 0.5 mM aliquots, ranging between 1-3 mM and the sunlight exposure time ranged between 30 min to 3 h. In case of kaempferitrin, 1 mg/mL was sufficient to produce a narrow peak, representing silver nanoparticle formation. 90 min of sunlight exposure was ideal for increasing the sharpness in the UV-spectra, further increase in time reduced the absorbance, suggesting the possibility of degradation or agglomeration of silver particles (Table 1). The optical density (OD) value presented a sharp band for KF@AgNPs (λmax at 390 nm) (Fig. 1A–B). Further X-ray diffraction patterns and TEM data corroborated with the presence of crystalline AgNPs.

#### *Crotalaria juncea* extract and Kaempferitrin capped CuNPs

The parameters for optimization using *C. juncea* extracts/ metabolites for the synthesis of CuNPs were fixed as (i) *C. juncea* extract concentrations (ii) Copper precursor (C_4_H_6_CuO_4_) and (iii) Reducing agent ratio (RA1, RA2, RA3). Similarly, as done with AgNPs optimization, two of the parameters were kept constant and the desired parameter was varied at different concentrations. *C. juncea* extract concentration (2, 3 and 4 mg/mL) Vs Copper precursor (1 mM) and Reducing agent (µL/mL). From the overall optimization process, CJE@CuNPs was optimized at CJE concentration (2 mg/mL), cupric acetate (2 mM) and reducing agents’ combination [Ammonium hydroxide (NH_4_OH) and Hydrazine hydrate (H_6_N_2_O)] ratio at RA2 i.e. 10 and 20 µL/mL respectively was selected for synthesis and characterization (Table 1).

Kaempferitrin capped synthesis of CuNPs was better when 1 mg/mL of kaempferitrin was used than its lower concentrations, along with precursor CAM at 1 mM; with the reducing agents combination of 5 and 10 µL/mL respectively. When optimization was done for CAM, 2 mM was found to be good compared to 1 mM and 3 mM and lastly with reducing agents combinations (RA1, RA2 and RA3) were analyzed for better combination and found that RA1 was sufficient which is very low in concentration. Thus as an optimized factor, KF at 1 mg/mL, 2 mM of CAM and reducing agents at 5 and 10 µ L/mL was taken for synthesis, (Fig. 1C-D), (Table 1) stability analysis and characterization studies.

#### Stability studies of Silver and Copper Nanoparticles

**Figure 2:**
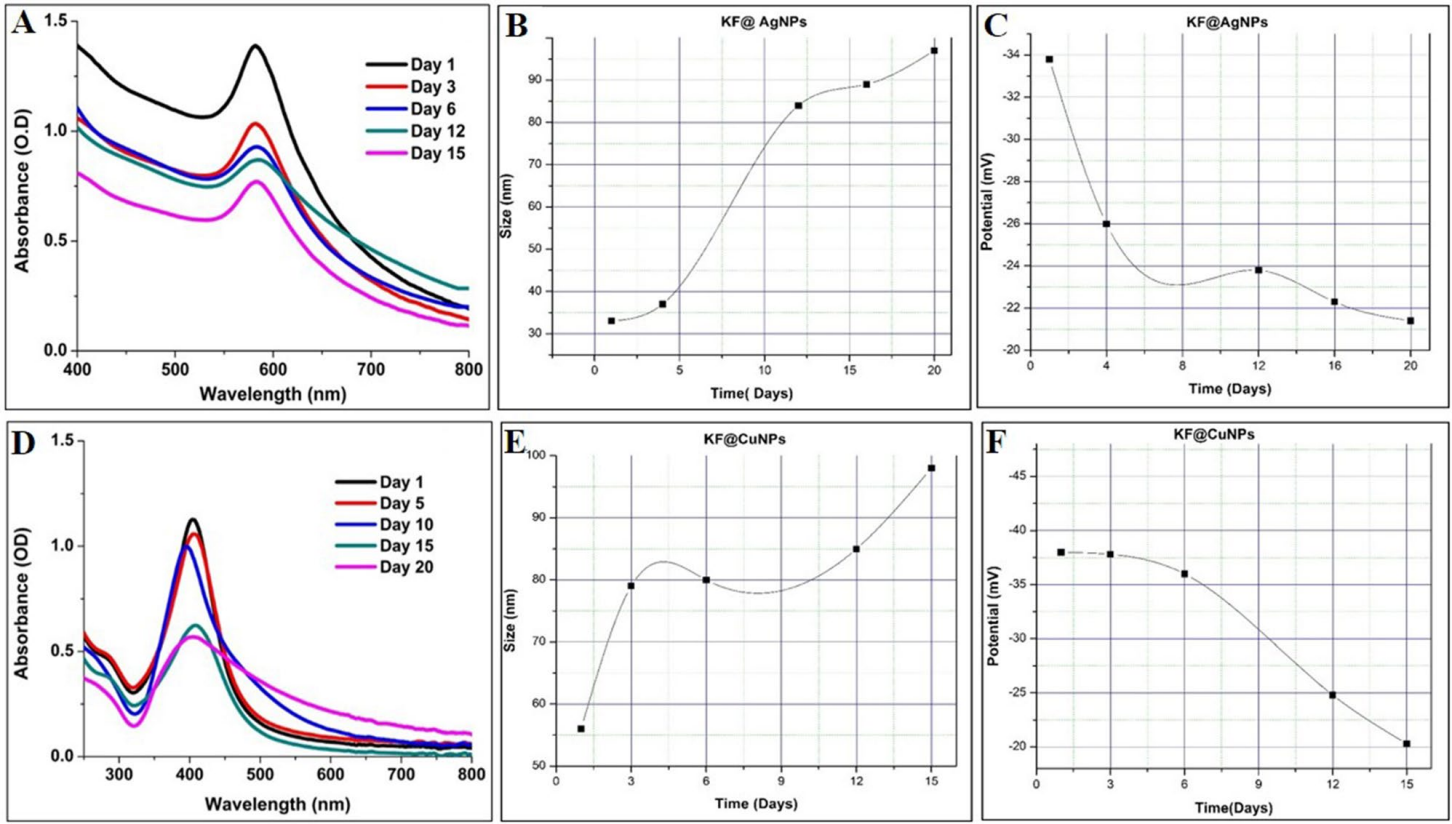
Stability studies on optimized Kaempferitrin-AgNPs/CuNPs (**A**–**D**) UV-spectrum **B**-(**E**) Zeta size profile (**C**–**F**) Zeta potential profile.

From the obtained optimization and dynamic light scattering data, it was evident that the stability of KF@AgNPs and KF@CuNPs was found to be stable for 20 and 15 days (Table 2). Therefore, further analysis for the stability of the synthesized Metallic NPs was conducted for KF@AgNPs and KF@CuNPs. The transition of the synthesized metallic NPs was observed during each time interval using Zeta sizer and potential determination (Fig. 2). Electrostatic repulsion of each individual particles depends on a large Zeta potential value above − 30 mV to + 30 mV that causes colloidal stability between the particles, whereas flocculation and aggregation occur when the value is small since van der Waals attractive forces act upon them resulting in physical instability^10^. There was a drastic increase in the size of KF@AgNPs from 33 to 97 nm and found to be stable for almost 20 days. Zeta Potential data showed a constant electro-kinetic potential in colloidal AgNPs from -33.8 to -21.4 mV (Table 2). KF@CuNPs portrayed the steady expansion of CuNPs size from 56 to 98 nm for 15 days (Table 2) and the zeta potential data indicated that the stability of CuNPs was consistent from − 38 to − 20.3 mV (Table 2). The decrease in the zeta potential energy over the course of time can be attributed to the increase in NPs size.

**Table 2:** DLS hydrodynamic diameter / Zeta Potential analysis of Kaempferitrin capped AgNPs/CuNPs.

Time (Days)		Zeta Potential (mV)	DLS hydrodynamic diameter (nm)	
Kaempferitrin Capped AgNPs				
1		− 33.8	33	
5		− 26.0	37	
10		− 23.8	84	
15		− 22.3	89	
20		− 21.4	97	
Kaempferitrin capped CuNPs				
1		− 38.0	56	
3		− 37.8	79	
6		− 36.0	80	
12		− 24.8	85	
15		− 20.3	98

### Characterization of CJE@AgNPs/CJE@CuNPs and KF@AgNPs/KF@CuNPs

**Figure 3:**
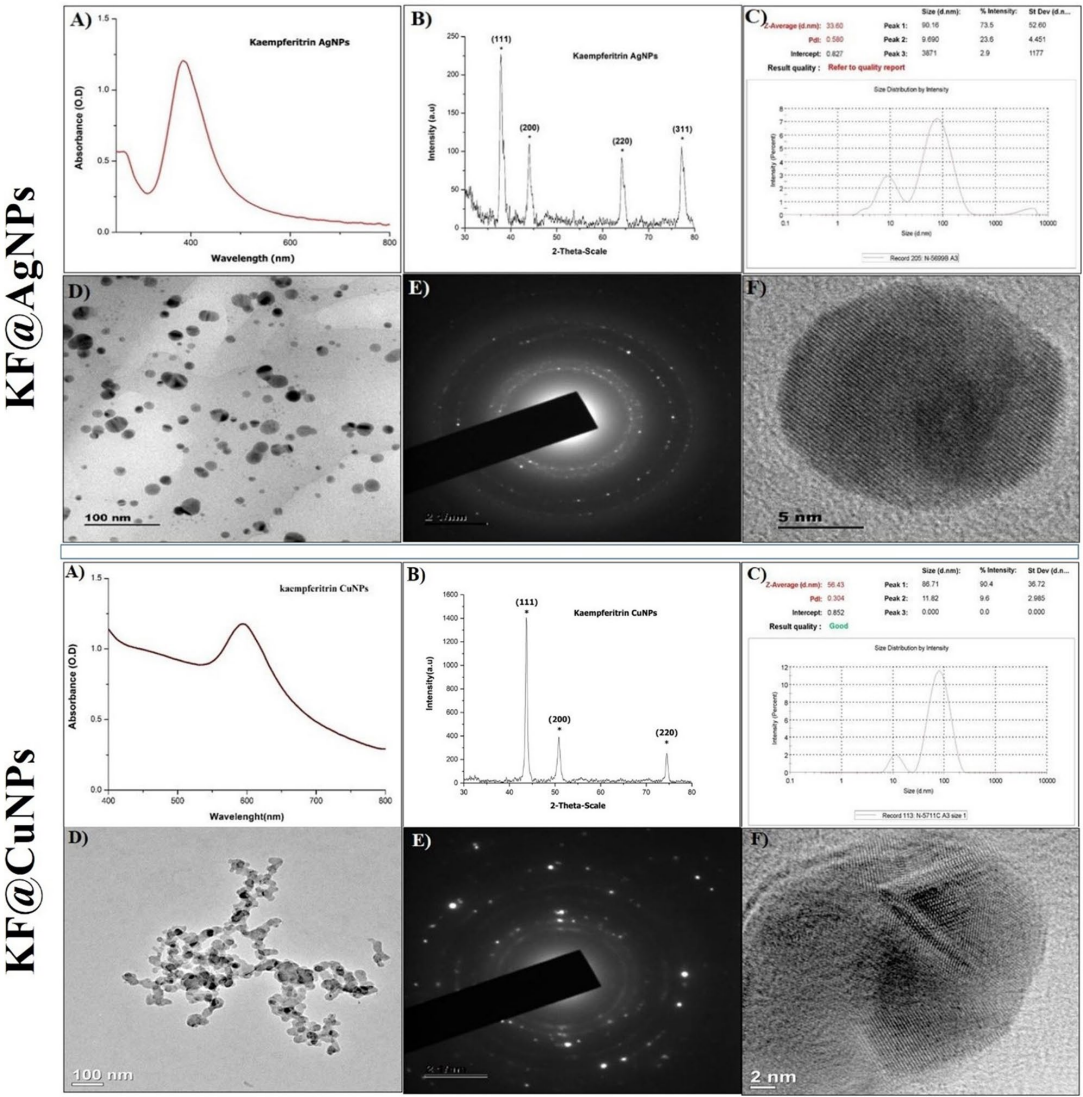
Physico-Chemical Characterization of Kaempferitrin AgNPs/CuNPs (**A**) UV Spectrum, (**B**) XRD, (**C**) Zeta Sizer, (**D**) TEM, (**E**) SAED, (**F**) HRTEM.

The CJE@AgNPs had an UV–Vis, λ_max_ at 415 nm, and the p-XRD 2θ values were at 38.03°, 44.08°, 64.47° and 77.27°, corresponding to the Miller indices (111), (200), (220) and (311) of silver, respectively. Zeta sizer analysis revealed a particle size range of ~ 64 nm (Fig. S4). KF@AgNPs absorbance was recorded at λ_max_ of 390 nm by UV–Vis spectroscopy. The presence of peaks at 2θ values 38.07°, 46.26°, 64.56° and 77.17° corresponds to (111), (200), (220) and (311) planes of KF@AgNPs, respectively. All the peaks in the XRD pattern can be readily indexed to a face-centered cubic structure of silver as per the available literature (JCPDS, File No. 4–0783). Zeta Sizer data estimated that the average sizes of AgNPs were at ~ 33 nm. TEM imaging revealed a regular spherical structure of the NPs formed. SAED TEM further revealed the crystalline structure of AgNPs and confirmed by HRTEM images (Fig. 3).

CJE@CuNPs recorded the λ_max_ at 590 using UV–Vis spectroscopy, the characteristic diffraction peaks of CJE@CuNPs were located at 43.01°, 50.11°, and 74.04° were observed. They correspond to the (111), (200), and (220) planes of the fcc structure of the zero-valent copper, respectively. An average size of ~ 70 nm of the CJE@CuNPs was found with the Zeta sizer experiments (Fig. S4). Absorbance from UV–Vis spectroscopy shows surface plasmon bands for KF@CuNPs at λ_max_ 590 nm, implying the existence of copper nanoparticles. The observed diffraction peaks were at 43.708° (111), 50.834° (200), and 74.508° (220) corresponding to the Miller indices (111), (200), and (220) respectively, indicating the crystalline nature of the CuNPs formed. Zeta sizer data for KF@CuNPs showed that the average sizes of the synthesized CuNPs of ~ 56 nm. TEM imaging revealed a regular spherical structure of CuNPs. SAED TEM further revealed the crystalline structure of CuNPs and confirmed by HRTEM images (Fig. 3).

### Effect of KF@AgNPs and KF@CuNPs on antibacterial/antibiofilm activity

The Minimum Inhibition Concentration (MIC) and Minimum Bacterial Concentration (MBC) of Kaempferitrin/ *C. juncea*, KF@AgNPs/KF@CuNPs and Doxycycline are depicted in Table 3. Kaempferitrin alone and not the extract of *C. juncea* had an effect on MRSA where MIC/MBC/MBIC was at 25/200/50 µg/mL respectively. However, when capped with Ag/Cu NPs enhanced antibacterial activity were observed. MICs/MBCs of KF@AgNPs were at 6.8/54 µg/mL, KF@CuNPs at 2/16 µg/mL. Minimum Biofilm Inhibitory concentration (MBIC) was evaluated using the Crystal violet assay, KF@AgNPs and KF@CuNPs were found to have MBIC of 3.4 and 1 µg/mL respectively. Thus, the data obtained indicated the antibiofilm effects of KF@AgNPs and KF@CuNPs were at sub-MIC values. Doxycycline MIC/MBC/MBIC was at 0.5/2/1 µg/mL. Therefore, further studies were performed using KF@Ag/Cu NPs. Thus, from the preliminary screening, KF@AgNPs and KF@CuNPs demonstrated true antibiofilm effect (devoid of antibacterial effect) and further studies on MRSA were explored.

**Table 3:** Screening of *C. juncea* extract /secondary metabolite and capped metallic nanoparticles against Methicillin-resistant *Staphylococcus aureus* (MRSA).

Secondary Metabolite (SM)/ Extract	SM/ Extract	SM capped AgNPs	SM capped CuNPs
	MICs/MBCs/MBICs (µg/mL)		
Kaempferitrin	25/200/50	6.75/54/3.37	2/16/1
*Crotalaria juncea*	> 400	54/216/108	32/128/32
Doxycycline	0.5/2/1	–	–

### Time kill kinetics

KF@CuNPs/AgNPs and Doxycycline caused a bactericidal effect against MRSA as monitored by plate counts at different time intervals from 0 to 24 h. KF@AgNPs/CuNPs caused a significant reduction of > 1.7/ > 1.9 log CFU, whereas Doxycycline treatment caused a substantial 1.6 log reduction in CFU (Fig. S5A).

### Determination of copper ions released

From the ARS-conjugation assay, we observed that the amount of copper ions released from KF@CuNPs is higher in the presence of MRSA. Whereas negligible release of Cu (II) ions was observed in its absence. In a reduced amount of bacterial count in media, the sensitivity increases. From the (Fig. S5B), it indicates, the bacterial cell surface aids to intensify the release of copper ions from KF@CuNPs during the time period of 24 h which might account for its enhanced antibacterial/antibiofilm potential against MRSA.

### Antibiofilm effects of Kaempferitrin capped AgNPs and CuNPs against MRSA

**Figure 4:**
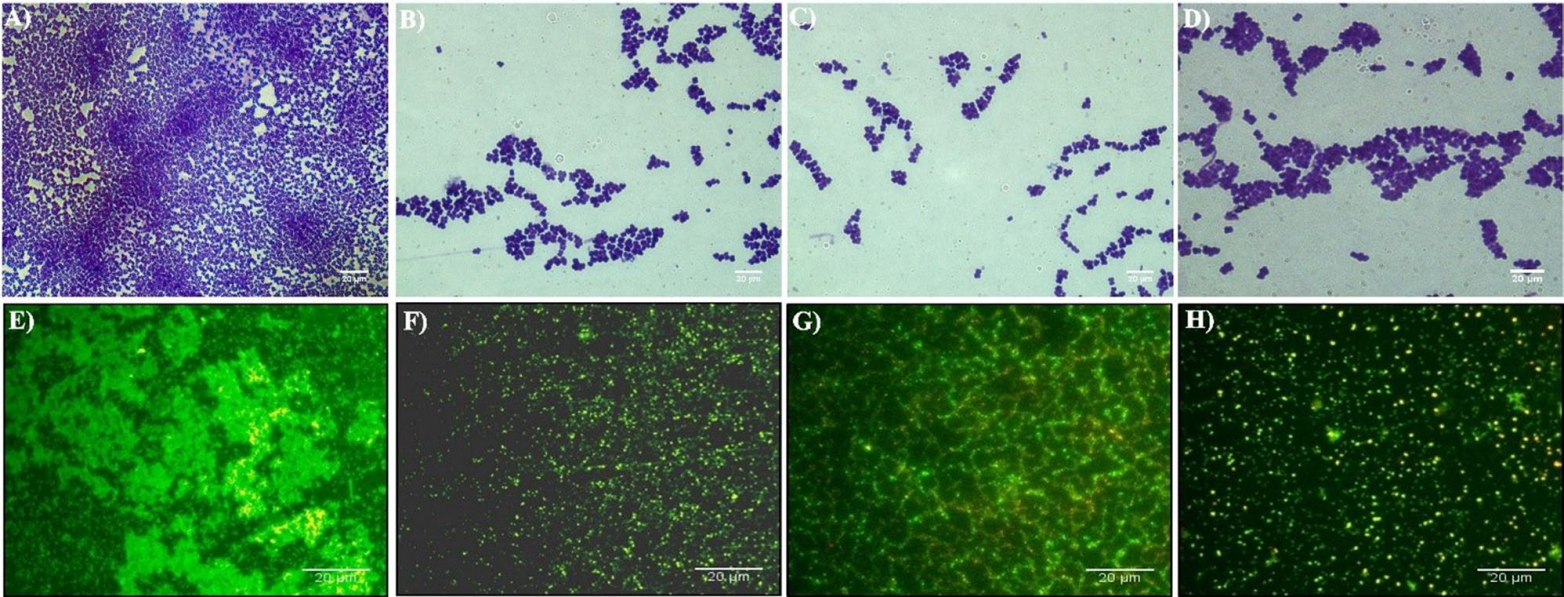
Crystal Violet and fluorescent imaging of Methicillin-resistant *S. aureus* biofilm. Crystal Violet staining of MRSA biofilms formed on glass surface **(A)** Untreated-MRSA biofilms **(B)** KF@AgNPs treated **(C)** KF@CuNPs treated and **(D)** Doxycycline treated. Fluorescence Live-Dead Imaging of biofilms formed on glass surface **(E)** Untreated, **(F)** KF@AgNPs, **(G)** KF@CuNPs, and **(H)** Doxycycline treated images. Live cells are indicated in green and dead cells in red, and the live/dead merged image appears yellow.

**Figure 5:**
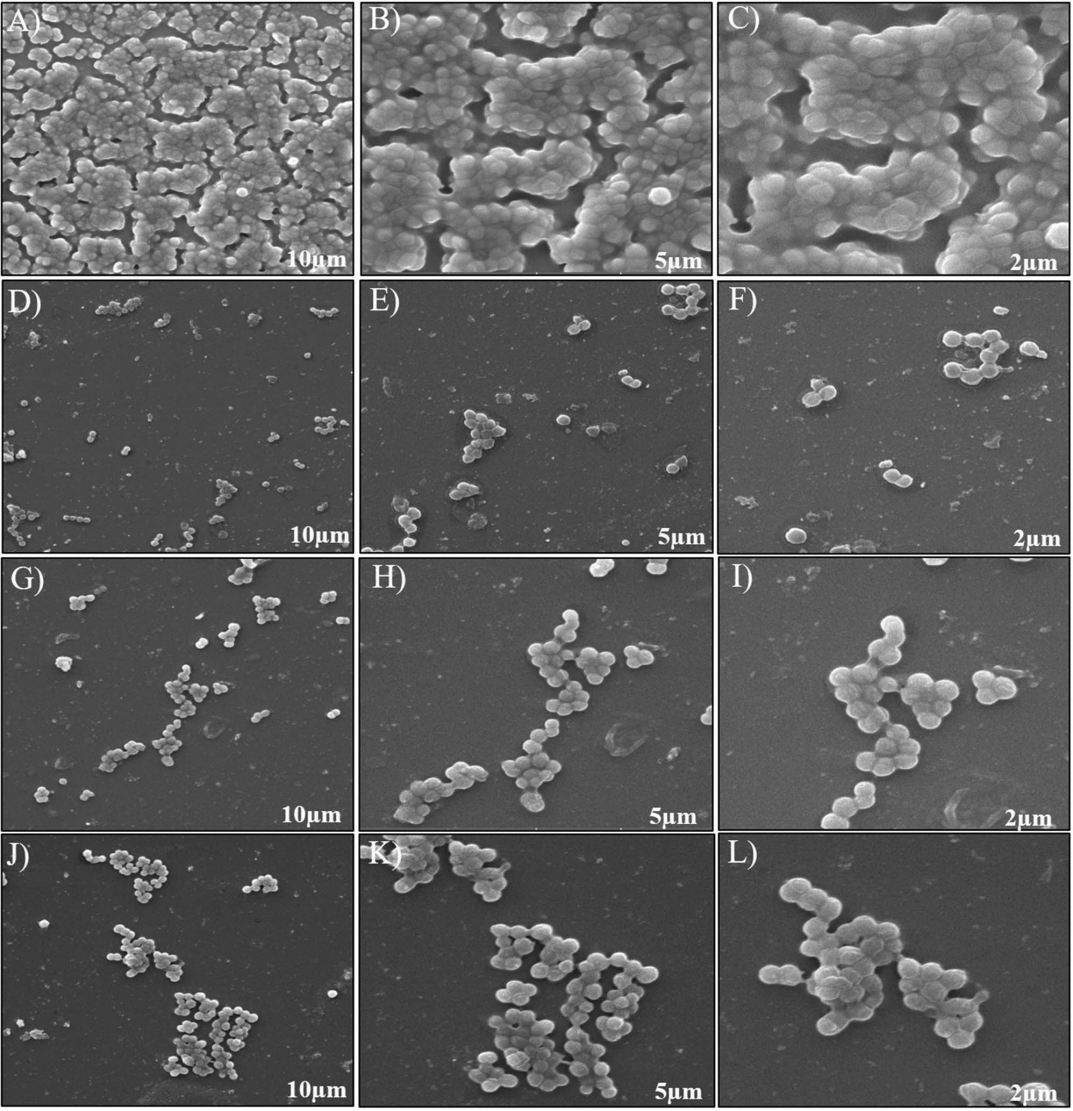
SEM imaging of MRSA biofilm treated with KF@AgNPs and KF@CuNPs. Unhindered colonization of untreated bacteria (**A**–**C**). KF@CuNPs treated (**D**–**F**), KF@AgNPs treated (**G**–**I**), and Doxycycline (**J**–**L**) treated images. Magnification @10 µm, 5 µm, 2 µm respectively.

Crystal Violet staining of treated and untreated biofilms shows inability to form biofilms in treatment groups KF@AgNPs, KF@CuNPs, and Doxycycline compared with the control (Fig. 4A-D) where biofilm formation is observed without any hindrance. From fluorescence live/dead staining, wherein apoptotic cells or membrane compromised cells emits red color fluorescence with PI staining whereas live cells were able to extrude PI and when stained with fluorescein diacetate it emits green fluorescence. Therefore, dead cells will appear as red and live cells will appear green. Dead cells that take up both the dyes will appear yellow in the merged image. Live dead imaging revealed treatment with KF@AgNPs, KF@CuNPs, and Doxycycline (Fig. 4E-H) were comparable and it resulted in reduced colony formation with fewer dead cells**.** Similarly, the SEM imaging revealed a near complete inhibition of MRSA biofilm formation in KF@AgNPs, KF@CuNPs and Doxycycline treatment groups (Fig. 5). Thus, from the imaging studies, the antibiofilm potential of KF@AgNPs and KF@CuNPs is evident.

### Mechanistic exploration of the antibiofilm effect

The data obtained from the imaging studies (Crystal Violet, Fluorescence Live/Dead, and SEM Imaging) reveals that KF@AgNPs/CuNPs, even at their Sub-MICs, concentration was able to retard biofilm formation. From these promising results, we further explored the possible mechanism involved in contributing to the antibiofilm potential of biogenic metal nanoparticles. An intact cell does not permeate Propidium Iodide (PI), therefore, accumulation of PI indicates enhanced membrane permeability caused due to nanoconjugate treatment^40^. CTAB, a well-known membrane-perturbing agent was taken as the positive control. Permeability index was expressed as the PI fluorescence ratio between untreated, and CTAB treated cells, which is depicted in percent. MRSA treatment with KF@AgNPs/KF@CuNPs resulted in a 30 to 45% rise in membrane permeability index in comparison to untreated control (Fig. S6A), which might account for antibiofilm potential^41^. Reactive oxygen species (ROS) damage the biological macromolecules and could account for the impaired biofilm formation. The ROS was quantified, and the results showed that there was no release of ROS in the KF@CuNPs treatment group; however, with the KF@AgNPs and Doxycycline intervention, the level of ROS release exceeded the normal untreated group levels. (Fig. S6B) Since ROS can also result in cell death and as we did not observe significant dead cells by live/dead staining, contribution of ROS to the antibiofilm potential of biogenic NPs is limited. BATH assay revealed that treatment with KF@AgNPs resulted in a 40% decline in Cell surface hydrophobicity, whereas KF@CuNPs caused 50% decline in CSH and there were no significant changes observed in the Doxycycline treated group, indicating that both AgNPs and CuNPs treatment might reduce adherence and hence might impair the biofilm forming ability of MRSA^42^ (Fig. S6C).

### Toxicity studies in Zebrafish model

As toxicity determination is a basic prerequisite, we have determined the toxicology parameters of biogenic MNPs in the zebrafish model by assaying brain acetylcholinesterase (AchE) and liver carboxyl esterases, AchE is an enzyme that catalyzes the breakdown of acetylcholine and other choline esters to accelerate neurotransmitter activity, therefore, can be used as a marker in neuronal toxicity studies. Inhibition of AchE functions causes prolonged motor neuron firing to alter animal response and behavior^43^ and liver carboxylesterase (CEs) activity plays a vital role in the detoxification of xenobiotics. Carboxylesterase belongs to the α/β hydrolase family that gets activated during xenobiotic stress and mediates the cleavage of esters and are considered major components of the liver detoxification system^44^. The toxicity of KF@AgNPs, KF@CuNPs, and Doxycycline on liver and brain enzyme profiles of Zebrafish were evaluated. There was no significant difference between the control and various treatment groups when analyzed for carboxylesterase through α-naphthol (Fig. S7A) and β-naphthol (Fig. S7B) in the liver. Similarly brain acetylcholinesterase level were determined for various groups and no significant difference among the treatment and control groups were observed (Fig. S7C), thus it can be inferred that biogenic nanoparticles do not alter brain and liver enzyme profiles and hence can be deemed to be non-toxic in zebrafish.

### Studies on histopathological changes in Zebrafish liver

**Figure 6:**
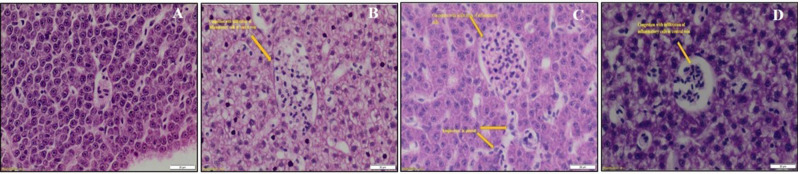
Histopathology changes in the liver of Zebrafish. Representative image of liver stained with Hematoxylin (purple color-nuclei) and eosin (pink color-cytoplasm). (**A**) Untreated liver (**B**) Doxycycline (**C**) KF@AgNPs and (**D**) KF@CuNPs treated.

Data obtained from the toxicity analysis for estimation of liver (α-naphthol and β-naphthol) enzyme level, depict no variation in the liver and brain enzyme levels when exposed to the treatment group, therefore further to extend these observations, the histopathological study on the zebrafish liver was performed to discern the effect of KF@Ag/Cu-NPs. The major challenge for drug development and clinical medicine is drug-induced liver injury. Zebrafish use similar pathways as humans to metabolize a wide range of hepatotoxic drugs when exposed^45^**. **As per the histological data, when treated with Doxycycline, congestion with infiltration of inflammatory cells in the central vein along with cellular swelling and disorganization of hepatocytes vein was observed (Fig. 6B). As for KF@AgNPs treated, the observation was that there was a lymphocyte infiltration in the sinusoid, congestion with an accumulation of inflammatory cells in the central vein, and overall normal liver architecture being preserved (Fig. 6C). The KF@CuNPs treated group also exhibited congestion with infiltration of inflammatory cells in the central vein, but a mild scattered inflammation was notedly observed (Fig. 6D), as compared to untreated control, depicts unaltered liver morphology (Fig. S8). Thus, histopathological analysis of metal nanoparticle treated liver exhibits mild inflammation but the enzyme profile reveals that biogenic metal nanoparticles are nontoxic.

### Zebrafish infection study

**Figure 7:**
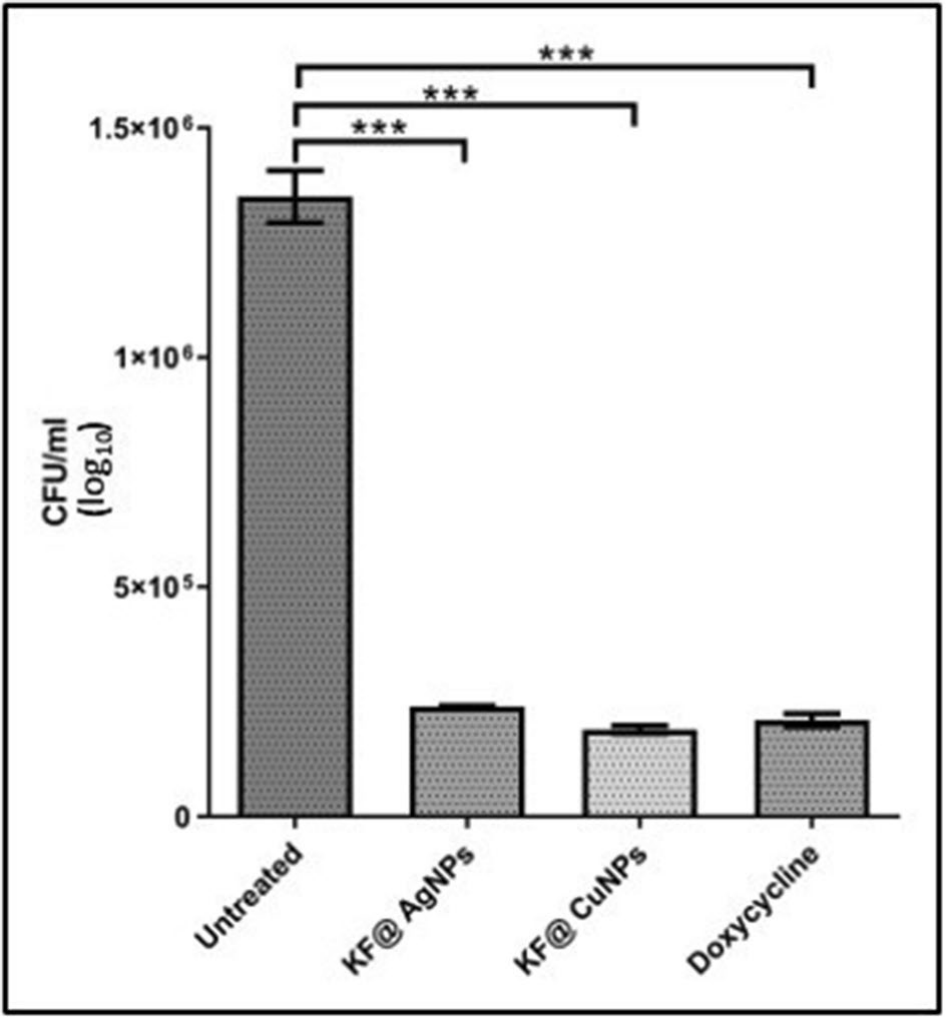
Infection study on Zebra fish model. Colony counts after treatment with KF@AgNPs, KF@CuNPS and Doxycycline. ***p < 0.0001.

Zebrafish (*Danio* *rerio*) by virtue of its genetic homology with humans is an ideal model system for investigating human infectious diseases models. It has unlocked new vistas in understanding pathogenesis and host–pathogen interactions. Several pathogenic zebrafish models have been generated using different species of bacteria, parasites, fungus, and viruses that are pathogenic to humans^46^. The promising data obtained after evaluating the antimicrobial/biofilm activity against MRSA from in vitro studies, encouraged us to further extend our investigation on in vivo zebrafish infection model and test the efficacy of biogenic AgNPs and CuNPs as a potent therapeutic agent. When MRSA infected Zebrafish were treated with KF@AgNPs, KF@CuNPs and Doxycycline, a drastic reduction in cell counts by ~ 1.8 log fold (Fig. 7), relative to untreated control was observed. An intriguing observation was that the decline in bacterial bioburden was noted at their sub-MIC concentration of AgNPs/CuNPs. It is likely that innate immunity of zebrafish mediated by macrophages and neutrophils aids in enhanced pathogen clearance in the presence of biogenic metal nanoparticles, which might account for decline in bioburden observed at sub-MIC concentrations.

## Discussion

Numerous reports suggest that the plant extracts acts as reducing and stabilizing agents in metallic nanoparticles synthesis^16,47^. These green synthesized nanoparticles were also proven as biomaterials, due to their biocompatibility and less toxicity^48^. Recent concern with the plant extract-based metal nanoparticles in bioactivity, is the variation with the chemical composition of plant extracts. The extract composition may vary due to various factors, which includes vegetation, seasonal variation, processing of plant material^49^. Nanoparticle size distribution is correlated with bioactivity^50,51^ and thus as an alternative to chemical synthesis of size tuned nanoparticle, pure phytochemical mediated (reducing and capping agent) will provide a safe and enhanced activity of the purified metabolites.

Plant phytochemicals-based synthesis of metallic nanoparticles are gaining importance for their biocompatibility and efficacy. Nano-conjugation with phytoconstituents usually results in formation of stable zero-valent metallic nanoparticles, which can result in an increased efficacy for the drug delivery^52^. In this regard, kaempferitrin, a flavonoid glycoside isolated from *C. juncea,* has been used in metal nanoconjugate fabrication. KF@AgNPs had a size range from 33 to 97 nm and found to be stable for almost 20 days, while KF@CuNPs ranged in 56–98 nm size and was stable for 15 days respectively.

MRSA, an opportunistic pathogen, has acquired a mobile genetic element, staphylococcal cassette chromosome *mec* (SCC*mec*), which plays an important role in staphylococci pathogenesis and confers resistance to almost all the β-lactam antibiotics. MRSA is a serious nosocomial pathogen and prevalence rate of MRSA is present in India^53^. WHO has announced that MRSA is a high priority pathogen that deserves immediate attention to develop novel antimicrobials or resistance modulatory agents. In such a scenario, finding an alternative to the existing drugs can be very challenging.

Based on the existing literature, *C. juncea* is reported to have antimicrobial properties, so with our investigation here in we reported the Kaempferitrin (flavonoid glycoside) as a major compound, obtained from the methanol extract through successive column chromatography and confirmed by NMR spectroscopy (Fig. S2 and S3). The antimicrobial and biofilm inhibition activities of the plant extract did not have a major impact as compared to isolated kaempferitrin, which were evidenced by the MIC/MBCs/MBICs values (Table 3). Thus, herein we have synthesized two metallic nanoparticles – Silver (AgNPs) and Copper (CuNPs), which are extensively used in medical appliances and their interference on the existing metal surfaces influences as a beneficiary. In order to reduce to zero-valent state [silver (Ag^+^) to Ag^0^ or copper (Cu^+^) to Cu^0^] the natural reducing agents (Phytochemicals) usage is known^54^ and for copper along with the phytochemicals, reducing agents such as Hydrazine hydrate were required, due to rapid oxidation tendency of copper.

The general mechanism for the synthesis of MNPs consists of three major stages, reduction of metal ions, clustering of NPs, and NPs growth^55^. During which the reducing agent donates an e^-^ to metal ions and are converted to high-energy state MNPs which will tend to return to its low surface energy conformation through aggregation. The presence of stabilizing and reducing agents restrict NPs from aggregation and results in the production of smaller NPs. Hydroxyl and carboxyl groups of polyphenols and polysaccharides, amine groups of protein from the plant extracts chelate metal ions and suppress Fenton reaction (superoxide-driven) which results in catalyzing the production of MNPs. In flavonoids, the hydroxyl group is majorly involved in metal binding activity^56^. According to some reports, the transformation process of tautomeric flavonoids from enol to keto form releases reactive hydrogen atoms that reduce metal ions to metallic NPs^57^.

Kaempferitrin, thus isolated from *C. juncea*, was explored for the possibility in synthesis of biogenic metallic nanoparticles. Optimization is a required process in all the operational systems to contribute to a systematic process. Various influencing factors such as extract/kaempferitrin concentration, metallic precursor (salts) and sunlight exposure time as photo-mediated in case of AgNPs or reducing agents as in CuNPs. With varying concentration of extract/ kaempferitrin, it was observed that the peak intensity and sharpness changes suggesting increase or decrease in concentration has a role in giving definition to nanoparticle formation (Fig. 1), similar trend was observed even in sunlight and it can be inferred that sunlight has major impact than the extract in regulating the formation of nanoparticles (Fig. 1). The precursor concentration (mM) was assessed for its importance in size and density of nanoparticle formation. CuNPs were formed by varying extract and with usage of minimal reducing agent volume (Fig. 1) Copper acetate concentration variation reduced the density of CuNPs formation suggesting low or higher concentration results in oxidation or aggregation and destabilizing the nanoparticles (Fig. 1).

During the synthesis of AgNPs, plant extract/secondary metabolite are mixed with AgNO_3,_ a transformation from pale to dark yellow was observed, indicating the AgNPs formation. Silver NPs in an aqueous solution emit light between 400 and 700 nm depending on their physical characteristics and exhibit strong surface plasmon resonance (SPR)^58^. UV–Vis spectroscopy analysis indicates the formation of AgNPs at 390–420 nm characteristic peak and CuNPs around 570–610 nm peak range. The presence of elemental zerovalent silver/copper and its crystalline nature was confirmed using X-ray diffraction (XRD) studies; TEM imaging revealed the structure of NPs (Fig. 3). SAED-TEM results further revealed the crystalline structure of metallic NPs and further corroborated with HRTEM images. Size of the biogenic nanoparticles synthesized from extract/metabolite capped had a ~ 60–79 nm using *C. juncea* extract (Fig. S4) and ~ 33–55 nm with Kaempferitrin capped nanoparticles (Fig. 3). Interestingly smaller size NPs were formed when capped with Kaempferitrin in contrast to the plant extract, which may be attributed to the presence of the hydroxyl group. While the photocatalytic mechanism for the synthesis of AgNPs is still not well understood, yet there are several proposed findings to establish an underlying mechanism. Previous studies demonstrated that the AgNPs photocatalytic activity under UV–vis illumination may be caused either by the production of Reactive Oxygen Species (ROS) or Ag^+^ from AgNPs^57^. Also in another proposed mechanism study, the electrons (e^-^) are excited to higher energy 5sp bands from 4d states of silver NPs in the presence of UV–vis illumination^40^.

Overall, from the data obtained, we found that the reduction time to transform Ag^+^ ions to AgNPs is higher in Kaempferitrin than the extract, which may be due to the presence of hydroxyl groups and conjugated double bonds in flavonoid, conferring high stability to the NPs^59^. Plant-mediated synthesis of MNPs has a major role as a reductant and stabilizing agent in metal nuclei due to its phytochemicals content. Among which flavonoids are assumed to release reactive Hydrogen (H) atoms during tautomeric alteration (enol to keto form) that reduce Cu (II) ions to form CuNPs. Metal ions with divalent/monovalent oxidation states get converted to Zero oxidation Cu nuclei and nucleation, the nuclei merged to form different physical shapes^60,61^.

The stability of NPs is another vital parameter depending on the area of application to be incorporated and varies based on synthesis technique, capping/stabilizing agents, and storage conditions. Comparatively, secondary metabolites act as better stabilizing agents than *C. juncea* extracts, indicating the low capping potential of the extract, and this can be attributed to the presence of other biomolecules-that may hinder the overall capping ability. Also, in general, biogenic AgNPs were stable for a longer period in comparison with CuNPs. This variation is due to the fact that CuNPs are extremely reactive resulting in rapid oxidation which makes it more unstable, on the other hand, Copper (Cu) gets easily converted to its oxide (CuO) state in pure CuNPs which can alter its mechanisms and physical properties. Hence there have been several approaches such as a protective layer or encapsulating with suitable organic agents on CuNPs to avoid oxidation^61^, again in correlation to its stability and size we observed that the smaller the size of the metallic nanoparticles longer is the stability, this phenomenon can be articulated with certain factors that affect the stability of nanoparticles. Stability in terms of aggregation, metal/metal oxide composition, nanoparticle shape, nanoparticle size, and surface chemistry^59^, specifically NPs based on size stability is an important parameter to an exploited physicochemical characteristic of the material. As the size of NPs decreases the repulsive forces between the particles rise beside the presence of surface chemistry further promotes the repulsive interactions^62^, subsequently to understand and provide add-on supportive information for the overall preliminary stability findings, KF@AgNPs and KF@CuNPs stability study was further conducted (Table 2). Wherein KF@AgNPs was stable for 20 days and Zeta potential data was noted from − 33.8 to − 21.4 mV, size of the AgNPs increased from 33 to 97 nm during the course of time. KF@CuNPs also shows the steady expansion of CuNPs size from 56 to 98 nm on the 15^th^ day and its Zeta potential data ranged from -38 to -24.8 mV. This finding correlates the stability (Zeta potential) of MNPs with its particle size.

The antimicrobial results of KF@AgNPs/KF@CuNPs against MRSA inferred that KF@AgNPs is more proactive than KF@CuNPs in curtailing bacterial growth with the MIC and MBIC of 6.8 ug/mL and 3.4 ug/mL respectively. From the time kill curve, (Fig. S5A) it was observed that bactericidal effects of both AgNPs and CuNPs were almost as similar to standard over a period of 24 h. From the alizarin test, it was confirmed that biogenic CuNPs release their cations (Cu^+^) into the growth medium (Fig. S5B) and these Cu^+^ ions using the electrostatic attraction stick on the cell wall of the bacterial cells or sometimes, these CuNPs can penetrate the bacteria through the membrane and lead to cell death^63,64^.

Biogenic nanoparticles were able to reduce biofilm formation at sub-MIC as is evident from crystal violet staining and Live/dead imaging (Fig. 4) and SEM analysis of biofilm (Fig. 5), suggesting that KF along with metallic ions reduces the biofilm growth as similar with other reports^65^. ROS studies and cell membrane perturbation studies (Fig.S6) indicate that KF@AgNPs, influences ROS generation at higher concentration (1X MIC). Reports suggest that silver ions released in media causes redox signaling and leads to cell death and reduced biofilm^66^. It correlates with other reports that biogenic nanoparticles reduce the hydrophobicity, which inhibits bacterial adhesion to surface, and reduce biofilm formation^67^ as seen in our study (Fig. S6).

The synthesized nanoparticles had good in vitro biofilm activity. To assess the impact of toxicity we used the zebrafish model and when analyzed for the liver/brain enzyme levels, no significant elevation of α and β-naphthol and acetylcholinesterase levels were observed with biogenic CuNPs/AgNPs treatment (Fig. S7), suggesting that the nano-conjugates are non-toxic^68^.

From the histological results, we observed that infiltration of inflammatory cells in the central vein along with cellular swelling and disorganization of hepatocytes vein were also found. In the presence of doxycycline and exposure to KF@AgNPs at 1X MIC caused lymphocytes infiltration in sinusoid, and congestion with an accumulation of inflammatory cells in central was observed. In KF@CuNPs treated group, congestion with infiltration of inflammatory cells in the central vein and mild scattered inflammation was observed (Fig. 6). An interesting observation was that the number of infiltration cells in the central vein was higher for KF@CuNPs among the other treated groups (Fig.S8) and this phenomenon may be due to CuNPs have a higher Trojan horse-type mechanism (release of massive intracellular ions in presence of media) than AgNPs^63^. However, no morbidity or co-morbidity symptoms were observed and thus biogenic nanoparticles can be considered non-toxic.

For the zebrafish infection model, MIC levels of both biogenic CuNPs and AgNPs were employed, and the MRSA infected results reveal that the biogenic MNPs caused a drastic reduction in colony count. KF@CuNPs caused a > 1.8 log fold reduction, KF@AgNPs had a higher > 1.9 log reduction and Doxycycline caused a > 1.6 log reduction respectively (Fig. 7) proving that the Biogenic KF@Ag/Cu-NPs were having almost identical ability to curtail bacterial growth which is comparable to antibacterial effect of Doxycycline.

## Conclusion

Our present study deals with Kaempferitrin, a natural nutraceutical agent isolated from *C. juncea,* was employed for formation of KF@AgNPs and KF@CuNPs. Kaempferitrin capped NPs with desirable morphology were obtained, as evidenced by TEM and particle size analysis. The stability study on the MNPs were evidenced with zeta potential and KF@AgNPs were found to be stable for 20 days and the KF@CuNPs were stable for 15 days, with no oxidation into copper oxide form. The designed NPs were further tested for its antibacterial and antibiofilm activities and capped NPs displayed bacteriostatic and bactericidal effect against MRSA, but its ability to inhibit biofilm formation at sub MIC levels depicted, that antibiofilm effect is independent of the antibacterial effect. Crystal violet-stained light microscopy images, SEM and fluorescence live/dead images showed reduced colonization of the solid substratum in the group treated with KF@AgNPs/KF@CuNPs. Alizarin red test indicated that the treatment with CuNPs for 24 h, released Cu (II) in presence of MRSA, causing damage to the cell membrane, enhancing membrane permeability and reducing cell surface hydrophobicity. The release of ROS was observed only in KF@AgNPs, not in KF@CuNPs. From the in vivo zebrafish toxicity study, no significant changes were observed in brain and liver enzyme levels, which were further corroborated with the histopathology analysis. Histopathological data revealed that no severe damage had occurred when treated with KF@AgNPs and it was almost similar to a normal liver. With the in vivo infection study, a significant reduction in bacterial bioburden was observed with both the Kaempferitrin capped MNPs. From our present study, we thus conclude that Kaempferitrin when used to functionalize the AgNPs/CuNPs the antimicrobial and antibiofilm activity gets enhanced on MRSA, with KF@AgNPs being dominant over KF@CuNPs.

## Supplementary Information

Supplementary Information.

## Data Availability

Upon reasonable request can be obtained from the Corresponding author.
