# Supplementary material for: Metal nanoparticles functionalized with nutraceutical Kaempferitrin from edible Crotalaria juncea, exert potent antimicrobial and antibiofilm effects against Methicillin-resistant Staphylococcus aureus
Source: Sci Rep. 2022 Apr 29;12:7061. doi: 10.1038/s41598-022-11004-2 (PMC9055053; doi:10.1038/s41598-022-11004-2)
Supplement: Supplementary file 1 — Supplementary Information. [file 41598_2022_11004_MOESM1_ESM.docx]

**Metal nanoparticles functionalized with nutraceutical Kaempferitrin from edible *Crotalaria juncea*, exert potent antimicrobial and antibiofilm effects against against Methicillin-resistant *Staphylococcus aureus***

***Corresponding authors: Aravind Sivasubramanian,** [**arvi@biotech.sastra.edu**](mailto:arvi@biotech.sastra.edu)

**
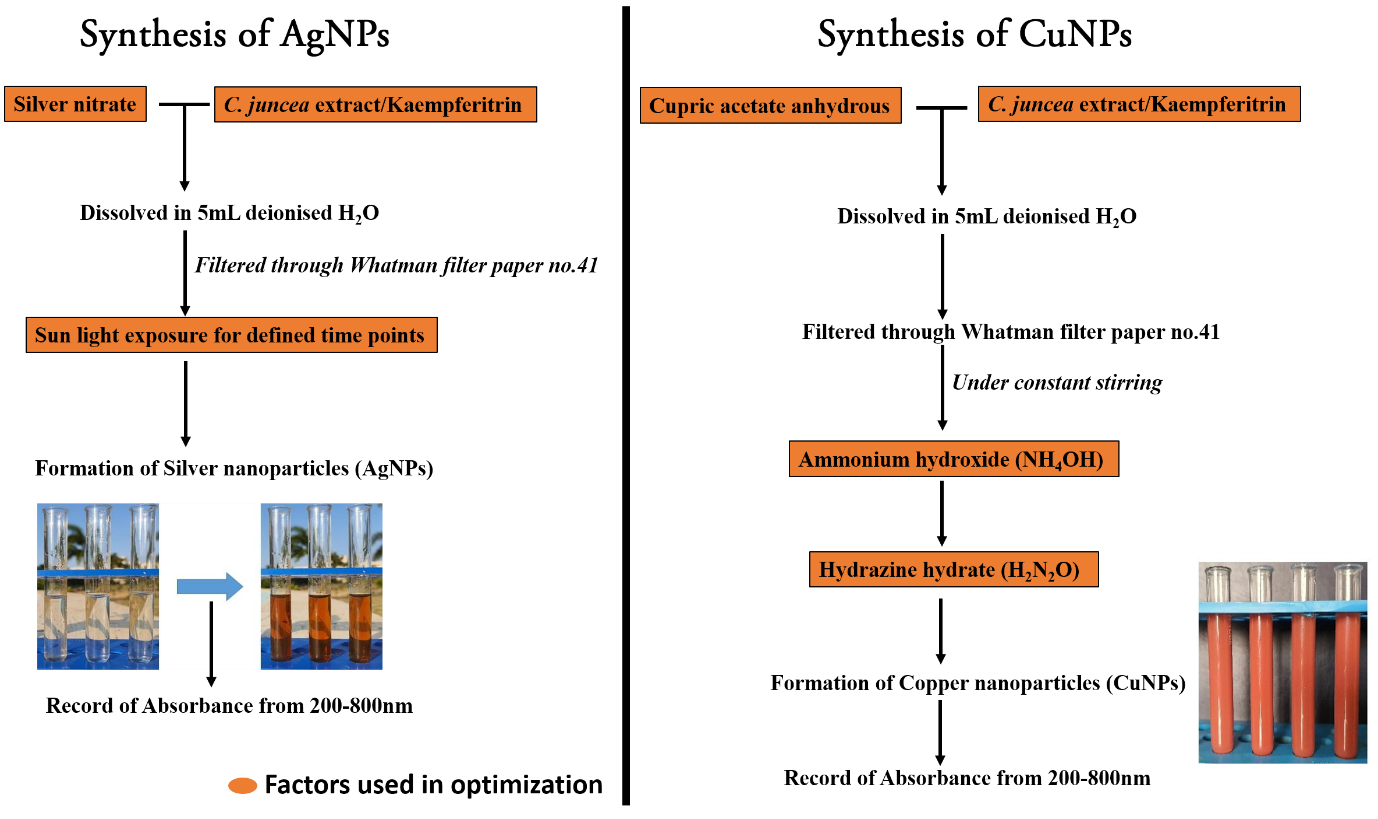
**

**Figure S1. Flow diagram for the process of synthesis and optimization of nanoparticles**

**
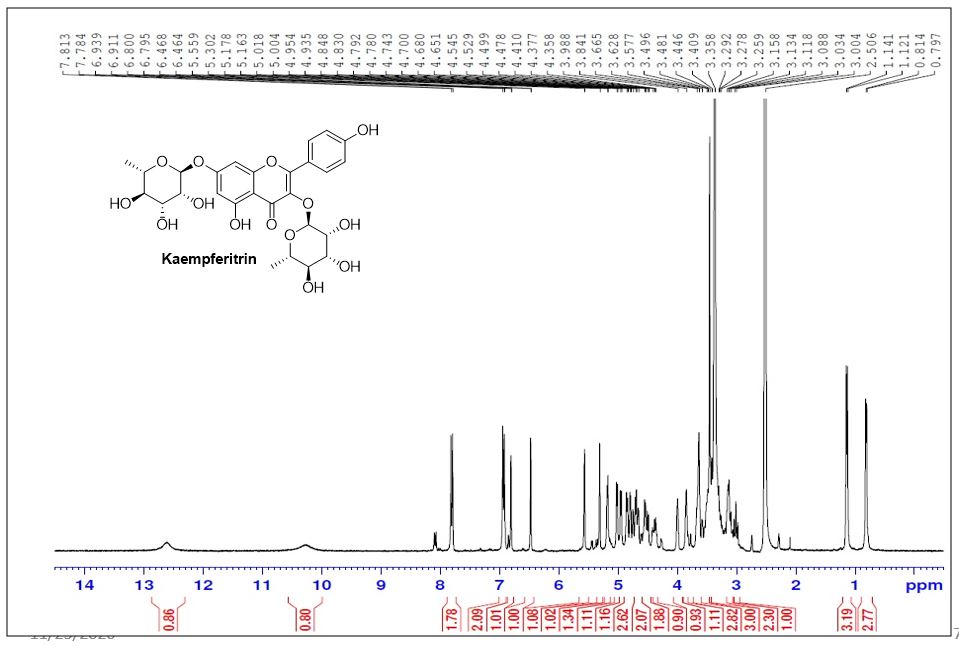
**

**Figure S2. ^1^H-NMR Spectrum of Kaempferitrin**

**
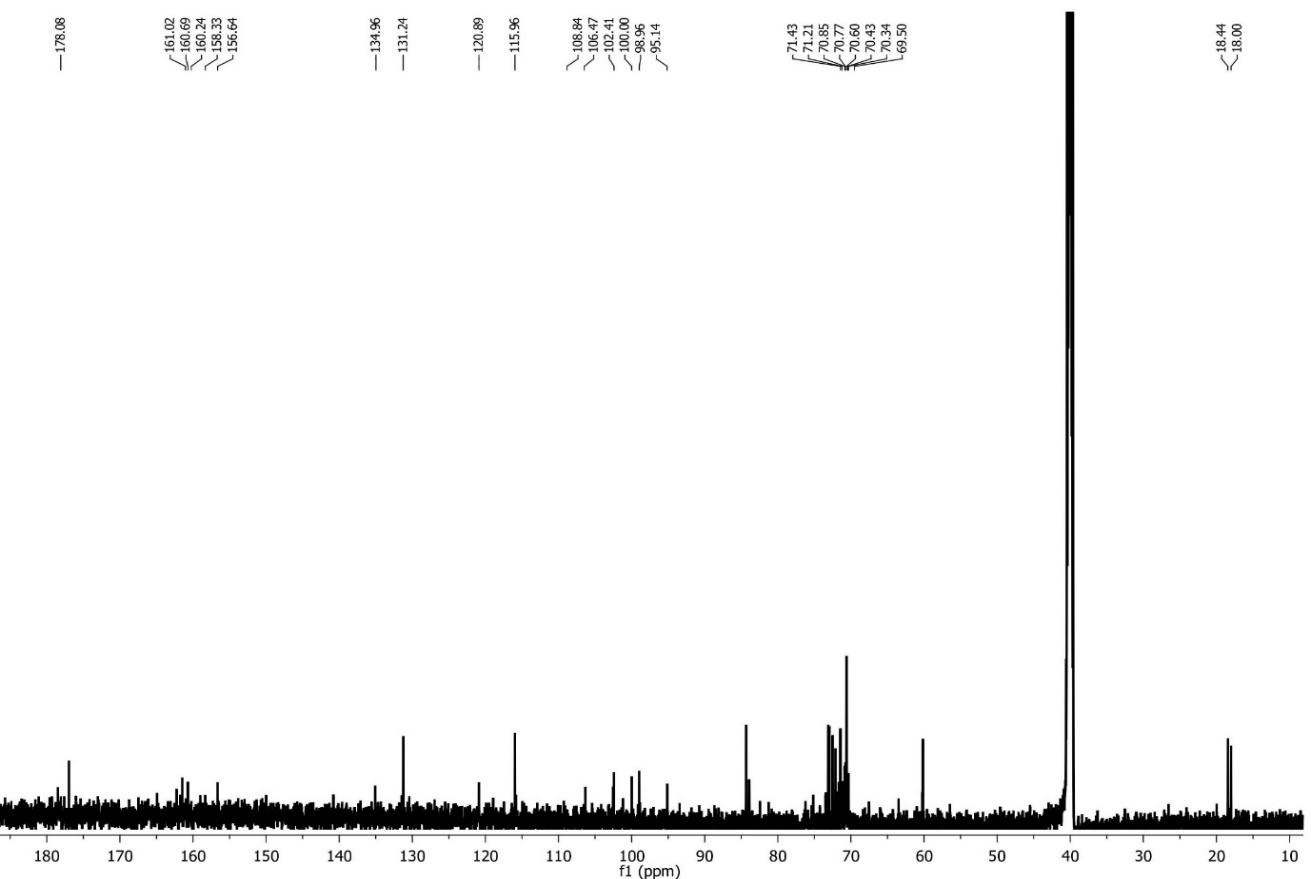
**

**Figure S3 ^13^C-NMR Spectrum of Kaempferitrin**

**
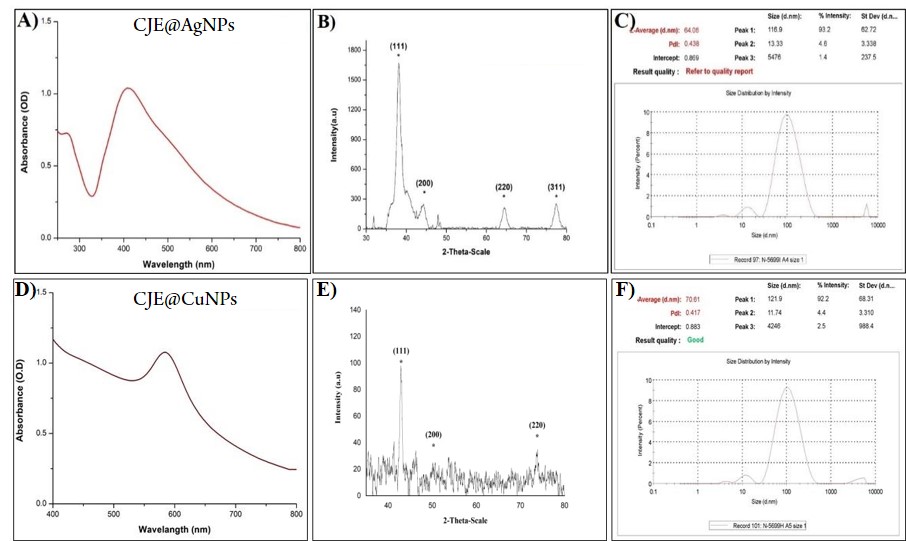
**

**Figure S4. Physico-Chemical Characterization of *Crotalaria juncea* AgNPs/CuNPs**

A-D) UV Spectrum, B-E) XRD, C-F) Zeta Sizer

**
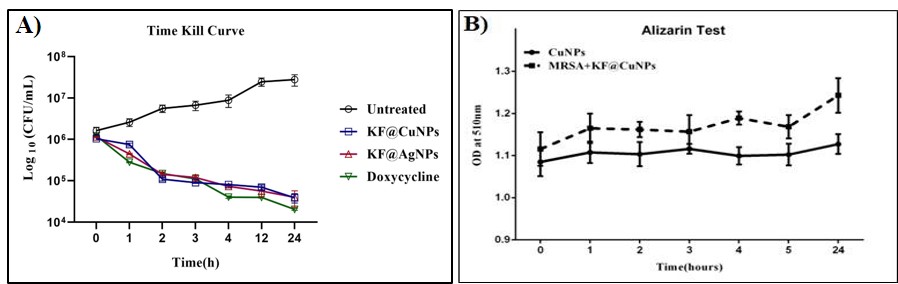
**

**Figure S5. (A) Time kill curve and (B) Alizarin Red Stain (ARS) conjugation test**

A). Time Kill analysis of MRSA in the presence of KF@CuNPs, and KF@AgNPs. Time course of

bacterial killing caused by various treatments was determined by plate count at various time intervals and

represented as log_10_ CFU/ml.

B). Time course of Cu(II) ion release from the media in the presence and absence of bacterial cells. The

released copper ions interact with alizarin red and exhibits intense red fluorescence which is quantified at

510 nm.


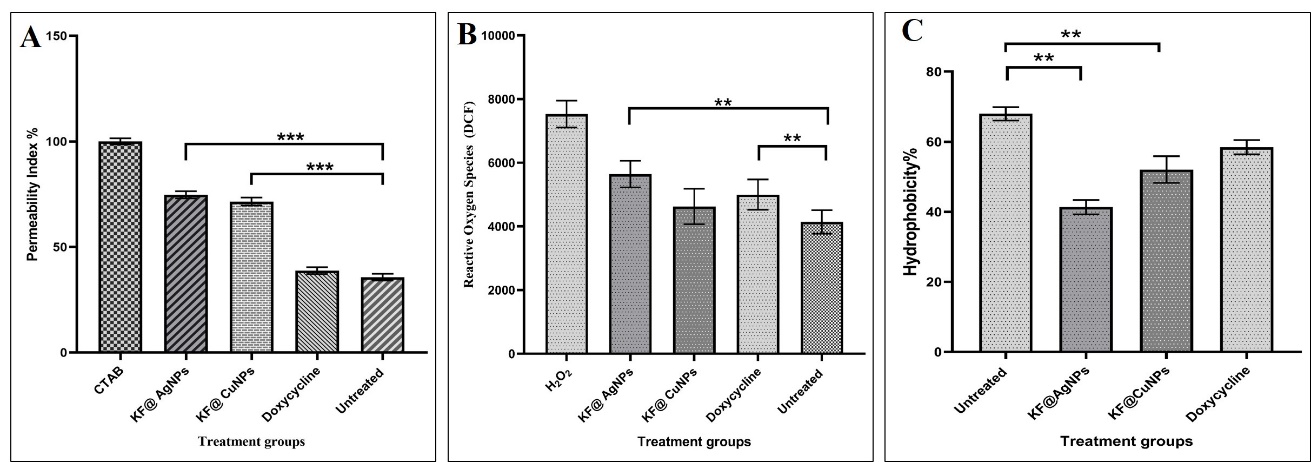


**Figure S6. Membrane perturbation, Reactive Oxygen Species and Hydrophobicity Assay**

**A) Membrane perturbation:** Changes in membrane permeability upon treatment with KF@AgNP, KF@CuNPs and Doxycycline respectively.

**B) Reactive Oxygen Species (ROS) generation:** Quantification of dichlorofluorescein, in comparison with positive control (H_2_O_2_), and treated group KF@AgNP, KF@CuNPs and Doxycycline respectively.

**C) Cell Surface Hydrophobicity:** Percentage of bacterial cell surface hydrophobicity upon treatment with KF@AgNPs and KF@CuNPs. Data represented as mean ± SD. (** p < 0.01 and *** p < 0.001)

**
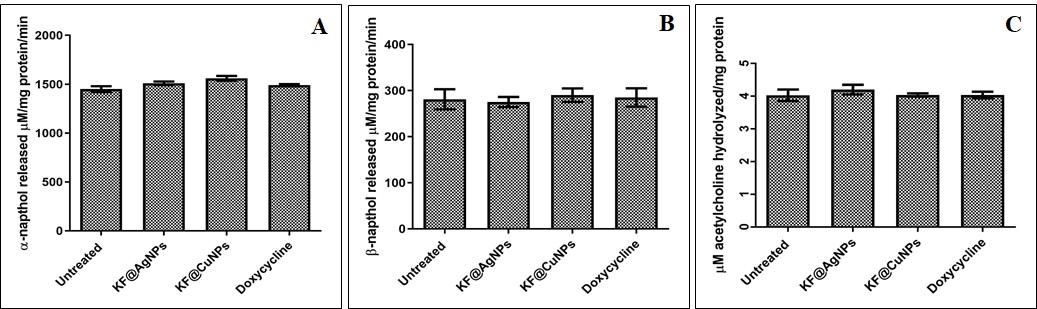
**

**Figure S7. Biogenic CuNPs effect on liver carboxylesterase and brain acetylcholinesterase activity**

KF@AgNPs/CuNPs and Doxycycline on liver carboxylesterase activity A)α-naphthol, B) β-naphthol and on the brain, C) acetylcholinesterase level.


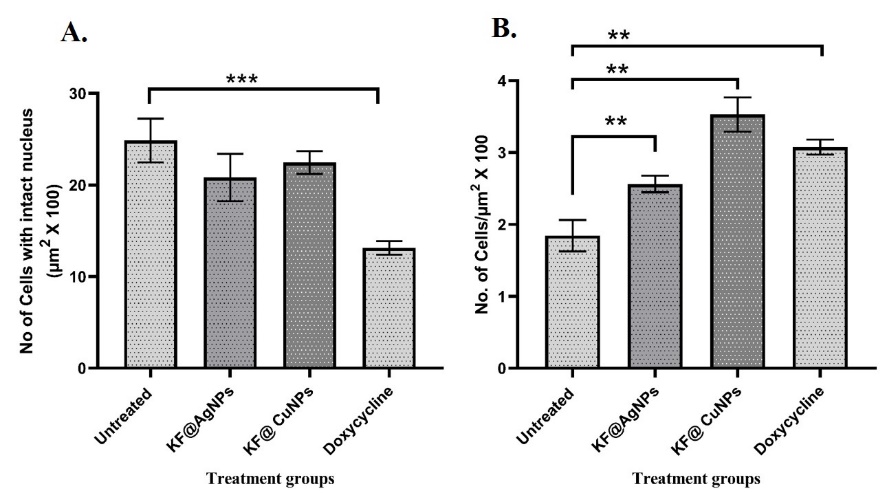


**Figure S8. Quantification of normal/ infiltrated cells in zebrafish liver**

A) Normal cells in the untreated, KF@AgNPs, KF@CuNPs and Doxycycline (n=5).

B) Infiltrated cells in the central vein compared with untreated and other treatment groups. **p<0.001 and ***p<0.001.
